# Extreme dominance of Earth-origin heavy ions in the intense ring current near the Earth during the May 2024 super geomagnetic storm

**DOI:** 10.1126/sciadv.aee1069

**Published:** 2026-06-26

**Authors:** Naritoshi Kitamura, Kazuhiro Yamamoto, Shoichiro Yokota, Satoshi Kasahara, Ayako Matsuoka, Kazushi Asamura, Yusuke Ebihara, Lynn M. Kistler, Kunihiro Keika, Atsuki Shinbori, Tomoaki Hori, Yoshizumi Miyoshi, Akimasa Ieda, Chae-Woo Jun, Mariko Teramoto, Masahito Nosé, Masafumi Hirahara, Kanako Seki, Nana Higashio, Iku Shinohara

**Affiliations:** ^1^ Institute for Space-Earth Environmental Research, Nagoya University, Nagoya, Japan.; ^2^ Department of Earth and Space Science, Graduate School of Science, The University of Osaka, Toyonaka, Japan.; ^3^ Department of Earth and Planetary Science, Graduate School of Science, The University of Tokyo, Tokyo, Japan.; ^4^ Data Analysis Center for Geomagnetism and Space Magnetism, Graduate School of Science, Kyoto University, Kyoto, Japan.; ^5^ Institute of Space and Astronautical Science, Japan Aerospace Exploration Agency, Sagamihara, Japan.; ^6^ Research Institute for Sustainable Humanosphere, Kyoto University, Uji, Japan.; ^7^ Institute for the Study of Earth, Oceans, and Space, University of New Hampshire, Durham, NH, USA.; ^8^ Kyung Hee University, Suwon, Korea.; ^9^ Department of Space Systems Engineering, Kyushu Institute of Technology, Kitakyushu, Japan.; ^10^ Graduate School of Data Science, Nagoya City University, Nagoya, Japan.; ^11^ Research Center for Advanced Science and Technology, The University of Tokyo, Tokyo, Japan.

## Abstract

Super geomagnetic storms are characterized by extreme intensification of the ring current in near-Earth space. The origin of the ions that carry the ring current is key to understanding its development. In situ measurements of ring current ions by the Arase satellite demonstrate an unprecedented dominance of heavy ions originating from the Earth during the May 2024 super geomagnetic storm, despite the high solar wind density. The solar wind, another expected source of ions, contributes little to the energy density of the ring current. This observational evidence highlights the critical role of ion supply processes from the Earth and transport in the magnetosphere in developing the ring current for the super geomagnetic storm. Furthermore, the super-intense ring current penetrated close to the Earth, strongly deforming the local geomagnetic field and driving unusual outward transport of electrons, which led to the loss of radiation belt electrons from the near-Earth region.

## Teaser

Earth-origin ions overwhelmingly dominated ring current ions in near-Earth space during the May 2024 super geomagnetic storm.

## INTRODUCTION

**Fig. 1.**
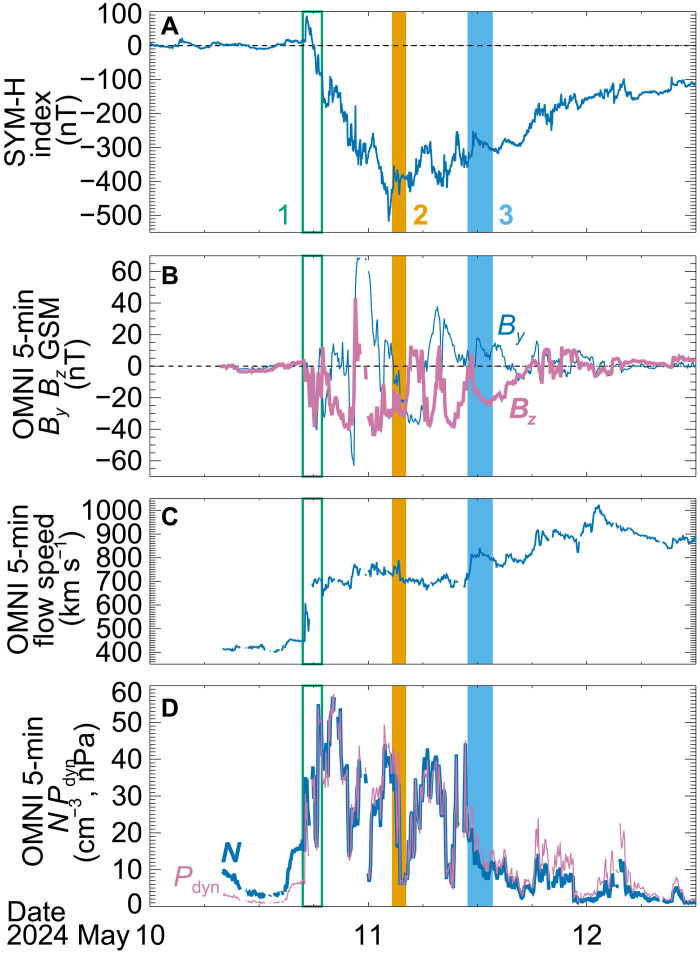
Solar wind conditions and geomagnetic index during the May 2024 super geomagnetic storm. (**A**) SYM-H geomagnetic disturbance index, which is widely used to represent the magnitude of geomagnetic storms with a temporal resolution of 1 min. (**B**) IMF [the *y* and *z* components in the geocentric solar magnetic (GSM) coordinates are shown as *B_y_* and *B_z_*]. (**C**) Solar wind flow velocity. (**D**) Number density (*N*) and dynamic pressure (*P*_dyn_) of the solar wind. Hatched regions denote the time intervals of Orbits 1 to 3 (duskside inner magnetosphere).

Geomagnetic storms are characterized by a decrease in the geomagnetic field intensity on the surface in the middle- and low-latitude regions of the Earth caused by the intensification of the westward ring current in near-Earth space (*1*, *2*). The minimum symmetric H (SYM-H) geomagnetic disturbance index (*3*) is commonly used as an indicator of the magnitude of geomagnetic storms. The minimum SYM-H of the May 2024 geomagnetic storm, −518 nT (Fig. 1A), is the second lowest for geomagnetic storms since 1981, when the SYM-H index became available. In situ observations of the ring current during magnetic storms of this magnitude are an unexplored frontier. Such unusually large geomagnetic storms are called super geomagnetic storms. Super geomagnetic storms are typically accompanied by drastic phenomena such as auroras extending into middle-latitude regions (*4*) and the disappearance and reformation of the electron radiation belt (*5*). Remarkably intense events can also severely affect modern infrastructure associated with daily life (*6*).

As the development of the westward ring current in the inner magnetosphere directly drives the growth of geomagnetic storms, revealing the characteristics of the ring current is fundamental to advance our understanding of geomagnetic storms. The ring current is carried mainly by high-energy ions, primarily with energies of tens of kiloelectron volts (keV), and the characteristics of such ions have been extensively studied (*1*, *7*–*28*). The westward ring current is mainly generated by an inward pressure gradient of the high-energy ions (*10*), and a high peak pressure is required to generate the strong pressure gradient that sustains the ring current for intense geomagnetic storms.

The composition of ring current ions is critically important in both growth and decay of the ring current and consequently for geomagnetic storms, because ion supply, acceleration, pitch angle scattering, and loss processes depend on ion species (*2*, *11*, *29*, *30*). Although the ring current was initially considered to predominantly consist of H^+^ (*1*), the finding of accelerated O^+^ in space changed this perspective (*31*). The ring current is carried by various ions originating from both the solar wind (primarily H^+^ and He^++^ ions) and the Earth’s ionosphere [H^+^, He^+^, and heavy ions with low-charge states; (*9*)] (*2*, *7*–*9*, *11*–*18*, *20*–*28*, *32*, *33*), in contrast to the energy driving geomagnetic storms, which originates from the disturbed solar wind. Studies on ion composition in the ring current predominantly focused on the proportion of each ion species in the local total energy density and its radial profile rather than on their contribution to the total pressure, as energy density is a simpler scalar quantity with a physical unit equivalent to that of pressure (a tensor) (*1*, *2*, *7*, *8*, *11*–*17*, *21*, *22*).

The characteristics of the super-intense ring current remain debated, although the abundance of O^+^ is known to increase during geomagnetic storms (*8*, *9*, *11*–*24*, *26*, *28*), particularly near solar maximum and/or during relatively large events (*11*–*13*, *16*, *17*). The lack of definitive conclusions in previous studies stems from limited measurements of ring current ions and the lack of concurrent solar wind measurements during super geomagnetic storms, despite the exceptional scientific importance of such events. There have been only two sets of in situ observations (Events 1 and 2) of ring current ions with species discrimination (*11*–*13*) during geomagnetic storms with a minimum SYM-H index below −250 nT, which is approximately half of the minimum SYM-H of the May 2024 storm studied here. This is attributed to the limited temporal coverage of in situ measurements of ring current ions until Solar Cycle 23, particularly near solar maxima. Hence, during other such geomagnetic storms, including the largest event in March 1989 (minimum SYM-H of −720 nT), satellites were not located in the inner magnetosphere near the peak of the events, or instruments for measuring ring current ions on satellites were unavailable. During Solar Cycle 24 (maximum around 2014), such events did not occur; the SYM-H index never dropped below −234 nT.

Event 1 is the February 1986 storm (~solar minimum) with a minimum SYM-H of −379 nT, and the maximum contribution of O^+^ and N^+^ to the total energy density (30 to 310 keV q^−1^) reached 59% (*11*). Event 2 is the March 1991 storm (~solar maximum) with a minimum SYM-H of −337 nT, and the contribution of O^+^ (N^+^ cannot be separated from O^+^) to the total energy density (40 to 420 keV q^−1^) reached ~65 to 75% (*12*, *13*).

As an extrapolation from these two earlier events, it is expected that more intense geomagnetic storms will be characterized by a substantially larger O^+^ proportion in the ring current. This expectation is also based on an observation of the high-energy component (9 to 210 keV q^−1^) of ions in the plasma sheet (PS) (not in the ring current) during a super geomagnetic storm in October 2003 (minimum SYM-H: −432 nT) (Event PS) (*34*). (As will be discussed in the final section, this energy range did not cover the range that is considered the most important at the peak of O^+^ energy density in the present study.)

In contrast, another expectation is that ions of solar wind origin may continue to contribute substantially (~30 to 60%) even near the peak of such events, owing to ion supply from the dense solar wind. This expectation is based on a simulation (without in situ observations of ring current ions) of the November 2003 storm (minimum SYM-H: −490 nT) driven by measured solar wind parameters (*33*). Although the available solar wind data during the event can be used as a driver of the magnetospheric simulation, a comparison with ion observations is not possible because of the lack of in situ observations of ring current ions. This super geomagnetic storm was characterized by a high solar wind density of ~10 to 30 cm^−3^ near the minimum SYM-H. This high density is a similar tendency to that of the May 2024 super geomagnetic storm, which is discussed in the next section.

Under these circumstances, it remains unclear whether O^+^ ions become overwhelmingly dominant even during super geomagnetic storms with high solar wind density or solar wind ions persist as a significant component of the ring current. Although a decrease in solar wind density may reduce the ion supply from the solar wind and contribute to an increase in the proportion of O^+^, the lack of concurrent solar wind measurements makes solar wind conditions that caused the dominance of O^+^ remain unclear even in Events 1, 2, and PS.

As the first opportunity to our knowledge to provide observational evidence on this issue, here, we report in situ observations of the ring current ions during the May 2024 super geomagnetic storm, demonstrating an extreme proportion of O^+^ in the ring current energy density and the critical importance of ion supply from the Earth even under high solar wind densities observed concurrently. This observational evidence highlights the critical role of ion supply and transport processes from the Earth in developing the ring current for the super geomagnetic storm. The radial distribution of ions is also an important characteristic of the ring current, and this event was the largest event ever achieved. We also report a substantial decrease in the local magnetic field intensity, even in the near-Earth region, caused by a super-intense current driven by terrestrial-origin ions transported close to the Earth. This decrease led to a considerable reduction in the radial gradient of magnetic field intensity, thereby affecting the transport of energetic charged particles even in the near-Earth region.

## RESULTS

### The May 2024 super geomagnetic storm

In May 2024, multiple coronal mass ejections from the Sun (*35*) triggered a super geomagnetic storm (minimum SYM-H: −518 nT; Fig. 1A), for which solar wind parameters were almost continuously available in the OMNI 5-min dataset (*36*). In addition to the development of the ring current, the storm caused various disturbances around the Earth (*37*–*43*). The energy driving the development of this storm was provided by fast solar wind (>700 km s^−1^) with a mostly southward-directed (negative *B*_z_) interplanetary magnetic field (IMF), which lasted until ~18 Universal Time (UT) on May 11 (Fig. 1, B and C). Even when the magnitude of southward *B_z_* decreased, the *y* component (*B_y_*) probably sustained the energy input owing to its large magnitude. If high-density solar wind ions (several tens of ions per cubic centimeter) (Fig. 1D) penetrate deep into the magnetosphere, then they can contribute to ring current ions (*44*). This is also plausible because ion density in the plasma sheet, which is the likely source of ions in the inner magnetosphere, is known to correlate with solar wind density (*45*, *46*).

Strong solar radiation heats the ionosphere, making it easier for terrestrial-origin (ionospheric) ions to flow out to the magnetosphere. The observed 10.7-cm solar flux (*F*_10.7_) (*47*) is a well-established indicator of solar radiation activity that strongly affects O^+^ ion outflow from the ionosphere (*48*). During ~11-year solar activity cycles, the yearly average *F*_10.7_ value varies from ~70 to ~200 solar flux units (1 sfu = 10^−22^ W m^−2^ Hz^−1^). The maximum values at solar maxima depend considerably on solar cycles, with maxima of 181.1 sfu (2001, cycle 23), 145.9 sfu (2014, cycle 24), and 191.6 sfu (2024, cycle 25). On May 7, the daily *F*_10.7_ exceeded 200 and remained elevated at 223.4, 213.7, and 221.8 sfu on May 10, 11, and 12, respectively.

### Ring current ion measurements

During the May 2024 event, the Arase satellite (*49*) successfully measured the energy density profile of ring current ions with species discrimination. Orbit Level-2 data (*50*) provided the orbital information, including *L*-shell (*L*_M_) values (*51*), which indicate the geocentric distance at the magnetic equator of the magnetic field line at the satellite location.

**Fig. 2.**
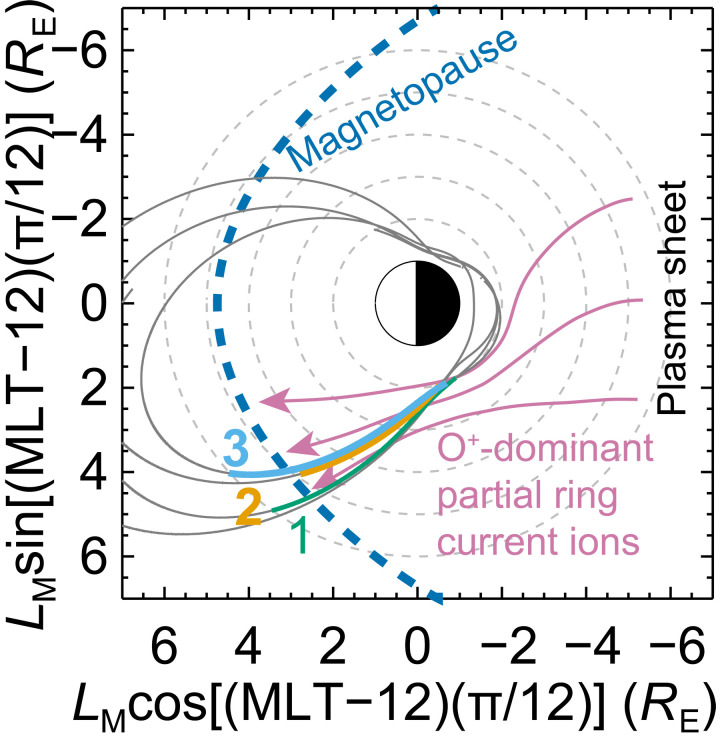
Orbit of the Arase satellite and schematic representation of ion drift motion. The trajectory of Arase is shown in the *L*_M_ and magnetic local time (MLT) plane. The highlighted segments with thick curves represent the three duskside passes (Orbits 1 to 3) during which ion composition and extremely high energy electrons were analyzed (Figs. 3 to 5 and figs. S2 to S5). The schematic representation of ion drift paths illustrates the westward motion of ions dominated by terrestrial-origin ions, which were injected from the nightside plasma sheet.

The medium-energy particle experiments–ion mass analyzer (MEP-i) (*52*, *53*) on Arase is well suited for observing ring current ions, particularly from the main phase to the early recovery phase (*11*, *21*, *22*). In the present study, we focused on duskside magnetosphere observations, for which MEP-i Normal mode data (see Materials and Methods) are available for the region near the magnetic equator despite an orbital inclination of ~31° (Fig. 2 and fig. S1). Furthermore, the duskside inner magnetosphere is suitable for observing ring current ions that drift westward from their probable upstream region, the nightside magnetosphere (plasma sheet) (Fig. 2), even when the ring current becomes strongly asymmetric in magnetic local time during the main phase of geomagnetic storms (*24*, *28*, *54*, *55*). By integrating the distribution functions from Normal mode observations, we obtained the energy densities (9.6 to 184.2 keV q^−1^) of H^+^, He^++^, He^+^, O^++^, O^+^, and molecular ions (a mixture of N_2_^+^, NO^+^, and O_2_^+^) (see Materials and Methods).

**Fig. 3.**
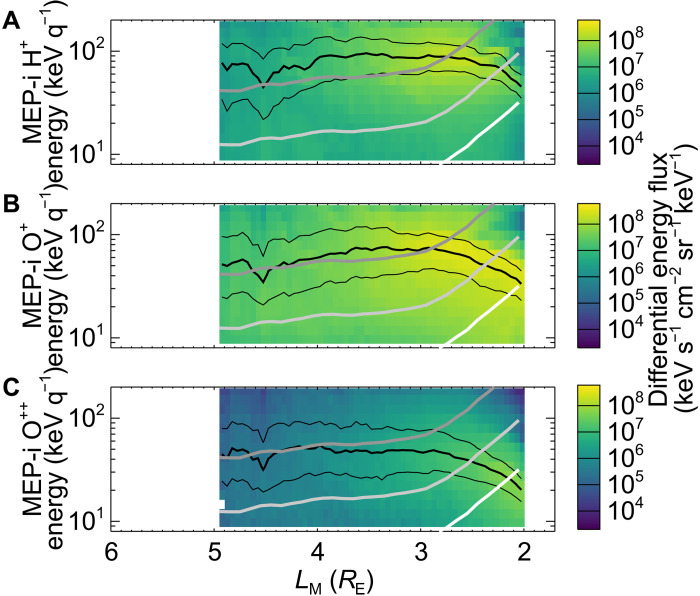
Energy versus the *L*_M_ spectra of differential energy fluxes of ions observed by the MEP-i (9.6 to 184.2 keV q^−1^) in Orbit 2. (**A**) H^+^. (**B**) O^+^. (**C**) O^++^. Black thin curves indicate cumulative energy density ratios of 0.25 and 0.75. The energy range between 0.25 and 0.75 is a good indicator of the energy range that contributes dominantly when the spacing is narrow. In such cases, the ratio of 0.5 (thick black curve) roughly indicates the center of the energy range. White, light gray, and gray curves indicate the first adiabatic invariants of 0.01, 0.03, and 0.1 keV q^−1^ nT^−1^, respectively, for ions with pitch angles of 90°.

Before examining the proportions of various ion species, it is important to verify that the energy range of MEP-i is appropriate for observing ring current ions even during this super geomagnetic storm, as this expectation was based on observations of smaller geomagnetic storms (*11*, *21*, *22*). The energy range of the ions primarily responsible for the total energy density can be approximated as the range between the contours in Fig. 3 and figs. S2 to S4 corresponding to cumulative energy density ratios of 0.25 and 0.75. Notably, this energy range for each ion species appeared around the middle of the energy range covered by MEP-i, particularly in Orbits 2 and 3, justifying that MEP-i covers the important energy range for ring current ions.

**Fig. 4.**
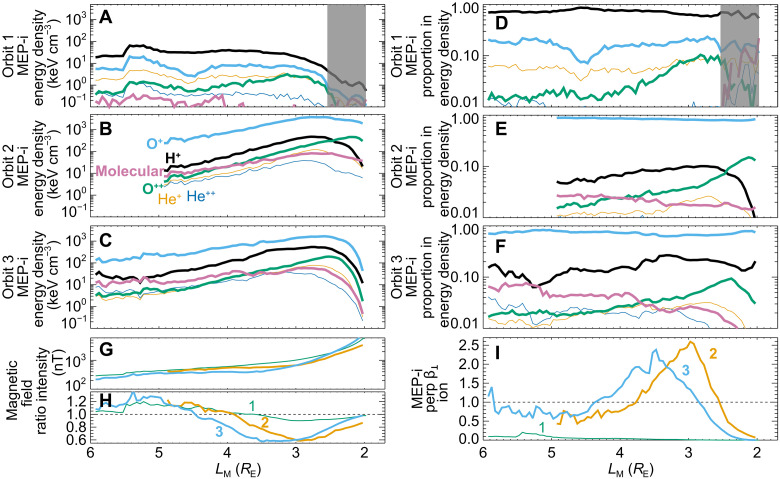
Spatial profiles of the energy densities of ions and magnetic field intensity. (**A** to **C**) Energy densities of ions observed by the MEP-i (9.6 to 184.2 keV q^−1^) in Orbits 1 to 3 (see Materials and Methods). (**D** to **F**) Proportion of each ion species in the total energy density in each *L*_M_ bin in Orbits 1 to 3. (**G**) Observed magnetic field intensity. (**H**) Magnetic field intensity ratio (observed / the IGRF model). (**I**) Ion $\beta_{⊥}$ (see Materials and Methods). The low-count interval in Orbit 1, where the energy density of the most abundant H^+^ was below 5 keV cm^−3^, was hatched. perp, perpendicular.

During the geomagnetic storm, solar wind with a large dynamic pressure arrived during Orbit 1 [~1706 UT at *L*_M_ ~ 5.5 Earth radii (*R*_E_)] (Figs. 1D and 4A and fig. S2). Although the SYM-H index began recovering before Orbit 2, the IMF during the inbound passes of Orbits 2 and 3 was still mostly oriented southward (Fig. 1B), driving the transport of ions from the plasma sheet into the inner magnetosphere and the ion loss process from the inner magnetosphere to the magnetopause (Fig. 2).

Relative to Orbit 1 (1648:00 to 1854:05 UT), when H^+^ dominates ring current ions as normally observed (*2*, *8*, *11*–*13*, *16*, *17*, *26*, *28*), the ion energy density observed in Orbits 2 (0236:00 to 0409:11 UT) and 3 (1054:00 to 1340:14 UT) remarkably increased owing to the arrival of ions from the nightside magnetosphere (Fig. 4, A to C). Terrestrial-origin ions (O^+^, O^++^, and molecular ions) became overwhelmingly dominant during Orbits 2 (~90%) and 3 (~70%), far exceeding the increase in solar wind–origin ions, even though the high-density solar wind was expected to supply a large amount of H^+^ into the ring current (Figs. 1D and 4, D and E). The large proportion of terrestrial-origin ions shown in Fig. 4 (D and E) were continuously observed in the magnetosphere; the extreme dominance of O^+^ was neither a local nor a short-lived (<1 hour) phenomenon.

**Fig. 5.**
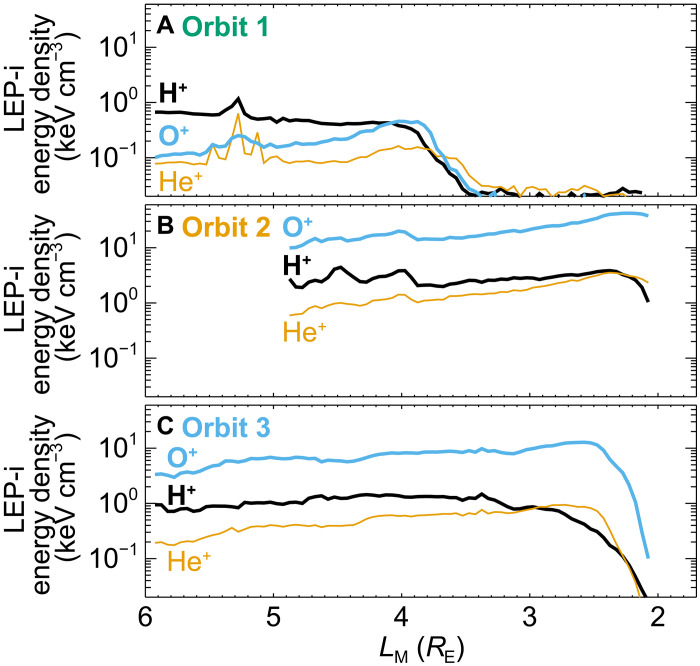
Spatial profiles of the energy densities of low-energy ions observed by the LEP-i. *L*_M_ profiles of energy densities of ions (0.1 to 8.2 keV q^−1^) (see Materials and Methods) in (**A**) Orbit 1, (**B**) Orbit 2, and (**C**) Orbit 3.

During Orbits 2 and 3, the energy density of O^+^ also dominated in the lower energy range (0.114 to 8.17 keV q^−1^) (Fig. 5), covered by the low-energy particle experiments–ion mass analyzer (LEP-i) (*56*, *57*) (see Materials and Methods). This range is below the energy range covered during Events 1 and 2. A much larger increase in the energy density of O^+^ compared with H^+^ and He^+^, particularly in the low *L*_M_ region, results in the dominance of O^+^ in this lower energy range. Although the characteristic of O^+^ dominance was similar, the contribution of lower-energy ions to the energy density was considerably lower than that of ions covered by MEP-i (<1% at *L*_M_ ~ 2.5 to 3.0 *R*_E_ near the peak of O^+^ energy densities in Orbit 2; Fig. 4B). Hence, we hereafter focus on ions in the energy range of MEP-i, covering six ion species.

In addition to O^+^, substantial amounts of O^++^ and molecular ions (N_2_^+^, NO^+^, and O_2_^+^) of terrestrial origin were observed by MEP-i. Although O^++^ has been considered a minor component of the ring current ions (*58*), in Orbit 2, the energy density of O^++^ exceeded that of H^+^ in the deep inner magnetosphere (*L*_M_ < 2.5 *R*_E_), and O^++^ became the second most abundant ion among the six species analyzed (Fig. 4, A and B). Furthermore, the tendency of O^++^ to be more abundant at low *L*_M_ is probably a feature specific to super geomagnetic storms, which are characterized by enhanced energy density in the deep inner magnetosphere.

The energy density of molecular ions also increased drastically after Orbit 1 (Fig. 4, A to F). Their proportion tended to be slightly higher in the larger *L*_M_ region in Orbits 2 and 3, in contrast to the O^++^ proportion of O^++^. The energy density reached its maximum in Orbit 3 at *L*_M_ > 3.7 *R*_E_ with a time delay relative to O^+^ (which peaked in Orbit 2). Although the ratio of molecular ions relative to O^+^ was consistent with previously reported values (*25*), their energy density became comparable to that of H^+^ at *L*_M_ ~ 5.3 *R*_E_, primarily because the H^+^ contribution was unusually low.

In contrast to the heavy ion species, the proportion of H^+^ in the total energy density became unprecedentedly small, less than ~10% in Orbit 2, which was close to the proportions of minor ions of terrestrial origin discussed above (Fig. 4E). As the contributions of helium ions (He^+^ and He^++^) to the total energy density are very small (<4% in Orbit 2 and <6% in Orbit 3), the sums of the energy densities of H^+^, He^++^, and He^+^ were <14 and <32% of the total energy density in Orbits 2 and 3, respectively (Fig. 4, E and F). These represent the upper limits of the contributions of solar wind ions, as H^+^ is considered to be a mixture of solar wind and terrestrial origin (*27*) and only some He^+^ can also originate from the solar wind via a charge exchange reaction between He^++^, originating from the solar wind, and neutral H from the geocorona (*59*).

In Orbit 2, the energy spectrum differs slightly depending on the ion species near the peak of O^+^ energy densities (*L*_M_ ~ 2.5 to 3.0 *R*_E_) and the inner region. In this part, we focus on the three main ion species: H^+^, O^+^, and O^++^ (Fig. 3). The central energy per charge of ions primarily contributes to the total energy density can be approximated as the energy of the cumulative energy density ratio of 0.5 (thick black contour in Fig. 3). The central contributor of O^+^ was a component with energy of ~20 keV q^−1^ lower than that of H^+^. The central energy of O^+^ decreased from 70 keV q^−1^ (*L*_M_ ~ 3 *R*_E_) to 30 keV q^−1^ (*L*_M_ ~ 2 *R*_E_) in the innermost region, where O^+^ energy densities remained more than about half of the peak value. In this region, contributions from the lower energy range (O^+^ below ~30 to 40 keV q^−1^), which were not covered in the earlier observations (Events 1 and 2), became substantial.

Although the central contributors of O^++^ and O^+^ were in almost the same energy per charge range at *L*_M_ > 4 *R*_E_, that of O^++^ was a component with energy per charge about half of that of O^+^ at *L*_M_ < 3 *R*_E_. This indicates that the energy of O^++^ was nearly equivalent to that of O^+^. If a reaction that causes the loss of one charge of O^+^ with little or no change in energy occurs near the Earth, it may simultaneously account for the relatively high proportion of O^++^ energy density and the difference in energy per charge that is specific to this region.

### Deformation of the geomagnetic field

If the ring current significantly deforms the background magnetic field, it alters the drift orbit of energetic ions and electrons around the Earth (*60*, *61*). As the distribution of ring current ions during geomagnetic storms of this magnitude had never been observed, spatial distributions and the magnitude of the magnetic field deformation remained unexplored. In contrast to regions with a weak background magnetic field far from the Earth (*60*, *61*), it is difficult to significantly deform the magnetic field because the background field intensity is much stronger near the Earth, where ring current heavy ions increased during the super geomagnetic storm.

The magnetic field intensity observed with the magnetic field experiment (MGF) (*62*, *63*) decreased by ~40% compared with that calculated using the International Geomagnetic Reference Field (IGRF) 14th model (*64*) even close to the Earth (*L*_M_ ~ 3 *R*_E_, just outside of the peak of O^+^ energy densities in Orbits 2 and 3) (Fig. 4, G and H). This decrease substantially alters the spatial distribution of the magnetic field gradient, resulting in a considerable reduction in radial gradient of the magnetic field intensity (roughly along the direction of the orbital motion of Arase) just outside the energy density peak (Fig. 4G). Enhancements of ion $\beta_{⊥}$ (see Materials and Methods) in Orbits 2 and 3 (Fig 4I) indicate that the ring current ions dominated by O^+^ had sufficiently high pressure to deform the background magnetic field even close to the Earth in the afternoon magnetic local time sector.

## DISCUSSION

With the reduced radial gradient, charged particles experience negligible magnetic field gradient drift in the azimuthal direction. Hence, equatorially mirroring particles drift radially inward and/or outward more easily under the influence of the convection electric field down to *L*_M_ ~ 3.5 *R*_E_, the inner edge of the region with a small radial gradient of geomagnetic field intensity. The magnetic field deformation induced by the ions themselves not only enables deep penetration of ions drifting from the nightside plasma sheet but also facilitates the drift out loss of outer ring current ions to the dayside magnetopause (Fig. 2) (*65*).

This deformation also affects the transport of radiation belt electrons orbiting around the Earth. As highly energetic electrons with equatorial pitch angles near 90° drift almost along contours of magnetic field intensity, they are efficiently transported to the magnetopause and are expected to be lost from the magnetosphere once a contour of magnetic field intensity connects to the magnetopause. This can cause a disappearance of energetic electrons further inward than the magnetopause. Consistent with this expectation, Arase did observe a reduction (almost disappearance) in the fluxes of the energetic electrons in the radiation belt (>1 MeV) (fig. S5), which were measured with the extremely high-energy electron experiment (XEP) (*66*, *67*), at *L*_M_ > 3.5 *R*_E_ in the region with a small radial gradient of geomagnetic field intensity.

The transport pathway of the ions that carry the ring current and deform the background magnetic field, as discussed above, is of great interest. To constrain the transport pathway of ions, we discuss the energy of ions prior to their transport to the inner magnetosphere using the first adiabatic invariant for a pitch angle of 90° in Orbit 2 (near the peak of the geomagnetic storm). Based on the first adiabatic invariant and the magnetic field intensity, we can roughly estimate the energy of ions in the plasma sheet, which is the probable upstream region for ring current ions. O^+^ at ~0.03 to 0.1 keV q^−1^ nT^−1^ had the largest contribution near the peak of its energy density at *L*_M_ ~ 2.5 to 3.0 *R*_E_ (Figs. 3B and 4B), whereas O^++^ at ~0.01 keV q^−1^ nT^−1^ was the most important near the peak at *L*_M_ of ~2.2 *R*_E_ (Figs. 3C and 4B). If ions are adiabatically transported from the plasma sheet, the energy of ions can be estimated by multiplying these values with the local magnetic field intensity. If ions are additionally accelerated nonadiabatically during transport, this estimation overestimates the ion energy in the upstream regions. Hence, this estimate likely represents an upper limit for the ion energy. Assuming representative magnetic field intensities of 100 and 10 near the geostationary orbit and in the near-Earth plasma sheet, respectively (*29*), the corresponding (upper limit) energies of O^+^ are estimated to be ~3 to 10 keV q^−1^ and ~0.3 to 1 keV q^−1^, respectively. These energy ranges fall below those covered during Events 1, 2, and PS; there have been no O^+^ observations near the peak of super geomagnetic storms in those regions. O^+^ below these energies in the near-Earth plasma sheet and the transport pathway of O^+^ to that region will be important observational subjects for future satellite missions. This estimate suggests that there may be a cool O^+^ population, as the ion temperature is ~5 keV in the plasma sheet under fast solar wind conditions (*46*, *68*).

The small adiabatic invariant indicates that O^+^ likely did not pass through a weak magnetic field region. In a more distant plasma sheet, where the magnetic field intensity is weaker, the first adiabatic invariants would be too large unless O^+^ has low energy (<0.1 keV q^−1^). This energy is inconsistent with the requirement that ionospheric O^+^ must be sufficiently accelerated to reach the more distant plasma sheet (*69*) and instead implies the importance of transport pathways that allow O^+^ to be injected directly into the near-Earth plasma sheet: either very low-energy O^+^ outflow from the cusp/cleft ionosphere (*69*–*71*) or direct O^+^ injection from the nightside auroral zone (*69*, *72*, *73*).

As the ring current ions penetrated close to the Earth, the charge exchange lifetimes of ions with the neutral hydrogen geocorona (see Materials and Methods) decreased substantially (fig. S6). As reported, the plasmapause contracted to *L*_M_ ~ 1.5 *R*_E_ (*42*); therefore, we calculated the upper limit of the lifetime (for ions with an equatorial pitch angle of 90°) down to the plasmapause. Inside the energy density peak of O^+^ (*L*_M_ < 2.5 *R*_E_), O^+^ with central energies of ~30 to 60 keV q^−1^ (Fig. 3B) is significantly lost within the timescale of the main phase (~10 hours). Therefore, charge exchange loss of ions during transport and/or during the main phase, as well as during the recovery phase, is an important factor in understanding the ring current during super geomagnetic storms. Faster losses of H^+^ (<45 keV q^−1^) probably contributed to the extreme situation in which the contribution of H^+^ to the energy density dropped to only ~1% at *L*_M_ ~ 2 *R*_E_ in Orbit 2 (Fig. 4E). The limit to which magnetospheric plasma can penetrate is likely associated with the low-latitude limit of auroras (*74*); magnetospheric ions are expected to penetrate with electrons that cause auroral precipitation. During larger (extreme) geomagnetic storms, as auroras have been observed even at very low magnetic latitudes (*40*, *74*), many ring current ions are also expected to penetrate to *L*_M_ ~ 2 *R*_E_ or smaller. Consequently, compositional changes arising from charge exchange will also be fundamentally important even near the peak time of geomagnetic storms.

In summary, near the peak of the May 2024 super geomagnetic storm, the ring current ions were overwhelmingly dominated (>85%) by terrestrial-origin heavy ions (O^+^, O^++^, and molecular ions) in energy density. The extremely heavy ion–rich condition was observed continuously along the orbit down to *L*_M_ ~ 2.0 *R*_E_ (the inner limit of ion measurements) in the duskside magnetosphere, which is a suitable region for observing ring current ions that drift westward from the nightside magnetosphere (Fig. 2); the condition was related to neither a local nor short-lived (<1 hour) phenomenon. In contrast, the contribution of solar wind ions (primarily H^+^) to the total energy density in the ring current was small (<15%), despite the high solar wind densities observed. The proportion of H^+^ in the super-intense ring current became comparable only to that of minor ion species of terrestrial origin. The energy density of O^+^ in the ring current peaked at *L*_M_ ~ 2.5 to 3.0 *R*_E_. Charge exchange loss even within the timescale of transport and/or during the main phase becomes substantial in the inner part of the super-intense ring current. The heavy ion–rich ring current, with a substantial $\beta_{⊥}$ > 1 near the Earth, affects particle transport via changes in drift orbits by modifying the background magnetic field even in the near-Earth region. These findings demonstrate that the overwhelming dominance of terrestrial-origin ions highlights the central role of ionospheric outflow processes and the supply, transport, and acceleration of ions from the ionosphere, particularly O^+^, in driving the development of super geomagnetic storms.

## MATERIALS AND METHODS

### Normal mode observations of MEP-i and LEP-i

MEP-i and LEP-i are operated in either the Normal mode for obtaining the three-dimensional velocity distribution function or the time-of-flight (TOF) mode, which specializes in obtaining the details of the ion mass spectrum. In some orbits (slightly higher latitudes) in the prenoon sector after the passage of the apogee (~32,000 km altitude), the instruments were operated in TOF mode, whereas on the duskside, the data were acquired continuously in Normal mode. We calculated the energy density of ions by integrating the velocity distribution functions of ions obtained by MEP-i and LEP-i in their Normal mode observations, as described in more detail in the next section.

The MEP-i Normal mode data (*53*) provide the differential fluxes of 16 spin phases, 16 azimuthal channels, and 16 energy channels for H^+^, He^++^, He^+^, O^++^, O^+^(+N^+^), and a molecular ion group that includes N_2_^+^, NO^+^, and O_2_^+^. MEP-i covers a field of view of 4π steradian with azimuthal and polar angle intervals of 22.5° during a half spin of Arase. The temporal resolution of the distribution function data was 8 s (1 spin). At *L*_M_ < 4 *R*_E_, four of 16 spin phases were available during the time intervals studied in this study to reduce the amount of data transmitted to ground stations. The first energy channel of MEP-i is unusable for scientific analysis due to the varying sweeping voltage of the electrostatic analyzer (*52*). By integrating data across the remaining 15 energy channels, we obtained energy densities for each ion species that cover an energy per charge range of 9.6 to 184.2 keV q^−1^.

Although Normal mode observations cannot distinguish N^+^ from O^+^ (the contribution of N^+^ is included in O^+^), it does not affect the comparison of the importance of ions of solar wind origin to those of terrestrial origin, given that both N^+^ and O^+^ are terrestrial origin ions (*75*), and N^+^ is considered to be minor [~30% of O^+^ or lower during a geomagnetic storm; (*19*)]. Because N^+^ exchanges a charge with an H atom more rapidly (rapid loss) than O^+^ (*75*), charge exchanges with a dense geocorona consisting of H act toward decreasing the energy density ratio between N^+^ and O^+^, particularly in the near-Earth region.

Although TOF mode data have usually been used to analyze molecular ions (*25*), the large ion fluxes after Orbit 2 allowed for the analysis of molecular ions with Normal mode data. An increase in counts from Orbit 1 to Orbit 2 is not due to contamination by energetic electrons, given that the flux of energetic electrons (>1 MeV) observed with XEP (*66*, *67*) decreased compared to that observed in Orbit 1 (fig. S5), when molecular ion counts were low (fig. S2F) as usual (*25*). The possibility of large contamination by O^+^ can be ruled out because of different temporal and spatial variations of the energy density of molecular ions from that of O^+^ in Orbits 2 and 3 (figs. S3, E and F, and S4, E and F).

When differential fluxes of ions are small, the energy density ratio becomes unreliable due to noise, and the proportions of minor ion species to the total energy density can be overestimated; this does not affect our conclusions, as we focus on regions with large energy densities (~large fluxes). Nevertheless, to avoid misinterpretation, we hatched the region where the energy density of the most abundant H^+^ was below 1 keV cm^−3^ in Orbit 1 (Fig. 4, A and D).

LEP-i Normal mode data provide differential fluxes of 16 spin phases, 15 azimuthal channels, and 32 energy channels for H^+^, He^+^, and O^+^ (*56*, *57*). The temporal resolution of the distribution function data was 8 s (1 spin). At *L*_M_ < 4 *R*_E_, four-spin averaged data (32 s) were provided in the intervals studied in this study to reduce the amount of data transmitted to ground stations. Similarly to MEP-i, we calculated the energy density between 0.114 and 8.17 keV q^−1^ (between the 6th and 20th energy channels of LEP-i), which covers the energy range below that of MEP-i.

As the magnetosphere was compressed due to the large dynamic pressure of the solar wind (Fig. 1D), LEP-i was also used to identify the magnetosheath- or cusp-like low-energy H^+^ with large differential energy fluxes. The data in the *L*-shells in which such H^+^ were observed (probably in the low-latitude boundary layer, magnetosheath, or cusp) were excluded from the analysis (*L*_M_ > 4.9 *R*_E_ in Orbit 2).

### Calculation of energy densities of ions and ion β

With 8-s spin-averaged magnetic field data obtained by MGF (*62*, *63*), we calculated the pitch angle (α) for each spin phase, azimuthal, and energy channel. Pitch angle distributions of differential fluxes [J(E,α)] were obtained for each energy channel by sorting the differential fluxes of ions into pitch angle bins with 15° width (Δα) (12 pitch angle bins; α = 0° to 15°, 15° to 30°, ..., and 165° to 180°). We calculated the energy density of each ion species according to the following equations with the trapezoidal rule

$$\begin{array}{c} \epsilon_{s}=\\ \pi \int_{0}^{\infty }\sqrt{2m_{s}E}\left[\int_{0}^{\pi }J_{s}\left(E,\alpha \right)\text{sin}\alpha d\alpha \right]\mathrm{dE}\approx \sqrt{2m_{s}}\pi \sum_{i=0}^{13}\left[\sum_{j=0}^{11}\langle \sqrt{E_{i}}J_{s}\left(E_{i},\alpha_{j}\right)\text{sin}\alpha_{j}\rangle \Delta \alpha \right]\Delta E_{i} \end{array}$$

where $m_{s}$ is the mass of ions, and the subscript s represents the ion species, and

$$\begin{array}{c} \langle \sqrt{E_{i}}J_{s}\left(E_{i},\alpha_{j}\right)\text{sin}\alpha_{j}\rangle =\\ \frac{\sqrt{E_{i}}J_{s}\left(E_{i},\alpha_{j}\right)\text{sin}\alpha_{j}+\sqrt{E_{i+1}}J_{s}\left(E_{i+1},\alpha_{j}\right)\text{sin}\alpha_{j}+\sqrt{E_{i}}J_{s}\left(E_{i},\alpha_{j+1}\right)\text{sin}\alpha_{j+1}+\sqrt{E_{i+1}}J_{s}\left(E_{i+1},\alpha_{j+1}\right)\text{sin}\alpha_{j+1}}{4} \end{array}$$

$$\Delta E_{i}=E_{i+1}-E_{i}$$

The mass of O_2_^+^ was used as a representative value for the molecular ion group. In this expression, physical quantity values in the International System of Units are used: $m_{s}$ [km], $E_{i}$[J] = Zq [C] × 10^3^ × $E_{i}$ [keV q^−1^], and (10^−4^ × Zq [C] × 10^3^)^−1^ × J [ions s^−1^ cm^−2^ (keV q^−1^)^−1^ sr^−1^], where Z is the number of charges and q is the elementary charge.

The contribution of each $\Delta E_{i}$ to the total energy density ($\epsilon_{s,i}$) is expressed as below

$$\epsilon_{s,i}=\sqrt{2m_{s}}\pi \sum_{j=0}^{11}\langle \sqrt{E_{i}}J_{s}\left(E_{i},\alpha_{j}\right)\text{sin}\alpha_{j}\rangle \Delta \alpha \Delta E_{i}$$

The energy densities in [J m^−3^] were converted to those in [keV cm^−3^] and were averaged in *L*_M_ bins with 0.05 *R*_E_ width (Figs. 3 and 4, A to C).

The ion pressure perpendicular to the background magnetic field ($P_{\text{ion}⊥}$) was calculated with the equations

$$P_{\text{ion}⊥}=\sum_{s}^{H^{+},\;{\text{He}}^{++},\;{\text{He}}^{+},\;O^{++},\;O^{+},\;\text{Molecular}}P_{s⊥}$$

$$\begin{array}{c} P_{s⊥}=\\ \pi \int_{0}^{\infty }\sqrt{2m_{s}E}\left[\int_{0}^{\infty }J_{s}\left(E,\alpha \right){\text{sin}}^{3}\alpha d\alpha \right]dE\approx \sqrt{2m_{s}}\pi \sum_{i=0}^{13}\left[\sum_{j=0}^{11}\langle \sqrt{E_{i}}J_{s}\left(E_{i},\alpha_{j}\right){\text{sin}}^{3}\alpha_{j}\rangle \Delta \alpha \right]\Delta E_{i} \end{array}$$

where

$$\begin{array}{c} \langle \sqrt{E_{i}}J_{s}\left(E_{i},\alpha_{j}\right){\text{sin}}^{3}\alpha_{j}\rangle =\\ \frac{\sqrt{E_{i}}J_{s}\left(E_{i},\alpha_{j}\right){\text{sin}}^{3}\alpha_{j}+\sqrt{E_{i+1}}J_{s}\left(E_{i+1},\alpha_{j}\right){\text{sin}}^{3}\alpha_{j}+\sqrt{E_{i}}J_{s}\left(E_{i},\alpha_{j+1}\right){\text{sin}}^{3}\alpha_{j+1}+\sqrt{E_{i+1}}J_{s}\left(E_{i+1},\alpha_{j+1}\right){\text{sin}}^{3}\alpha_{j+1}}{4} \end{array}$$

We calculated ion $\beta_{⊥}$, which is the ratio between $P_{\text{ion}⊥}$ and the magnetic pressure ($P_{B}$)

$$P_{B}=\frac{B^{2}}{2\mu_{0}}$$

where B is the magnetic field intensity, and $\mu_{0}$ is the permeability of vacuum.

### Calculations of the charge exchange lifetime of H^+^ and O^+^

The charge exchange lifetime t was calculated as $t=1/\left(n_{H}\sigma_{s}v\right)$, where $n_{H}$ is the neutral hydrogen density, σ is the charge exchange cross section with neutral hydrogen for ion species s, and v is the relative velocity. The neutral hydrogen density was taken from a model based on Monte Carlo simulations with *F*_10.7_ = 230 sfu (*76*). We adopted the reported charge exchange cross sections for H^+^ (*77*) and O^+^ (*78*).
