# Supplementary material for: Extreme dominance of Earth-origin heavy ions in the intense ring current near the Earth during the May 2024 super geomagnetic storm
Source: Sci Adv. 2026 Jun 26;12(26):eaee1069. doi: 10.1126/sciadv.aee1069 (PMC13308607; doi:10.1126/sciadv.aee1069)
Supplement: Supplementary file 1 — Figs. S1 to S6 [file sciadv.aee1069_sm.pdf]

Science Advances
AAAS

Supplementary Materials for

Extreme dominance of Earth-origin heavy ions in the intense ring current near the Earth during the May 2024 super geomagnetic storm

Naritoshi Kitamura *et al.*

Corresponding author: Naritoshi Kitamura, naritoshi.kitamura@nagoya-u.jp

Sci. Adv. **12**, eaee1069
DOI: 10.1126/sciadv.aee1069

This PDF file includes:

Figs. S1 to S6

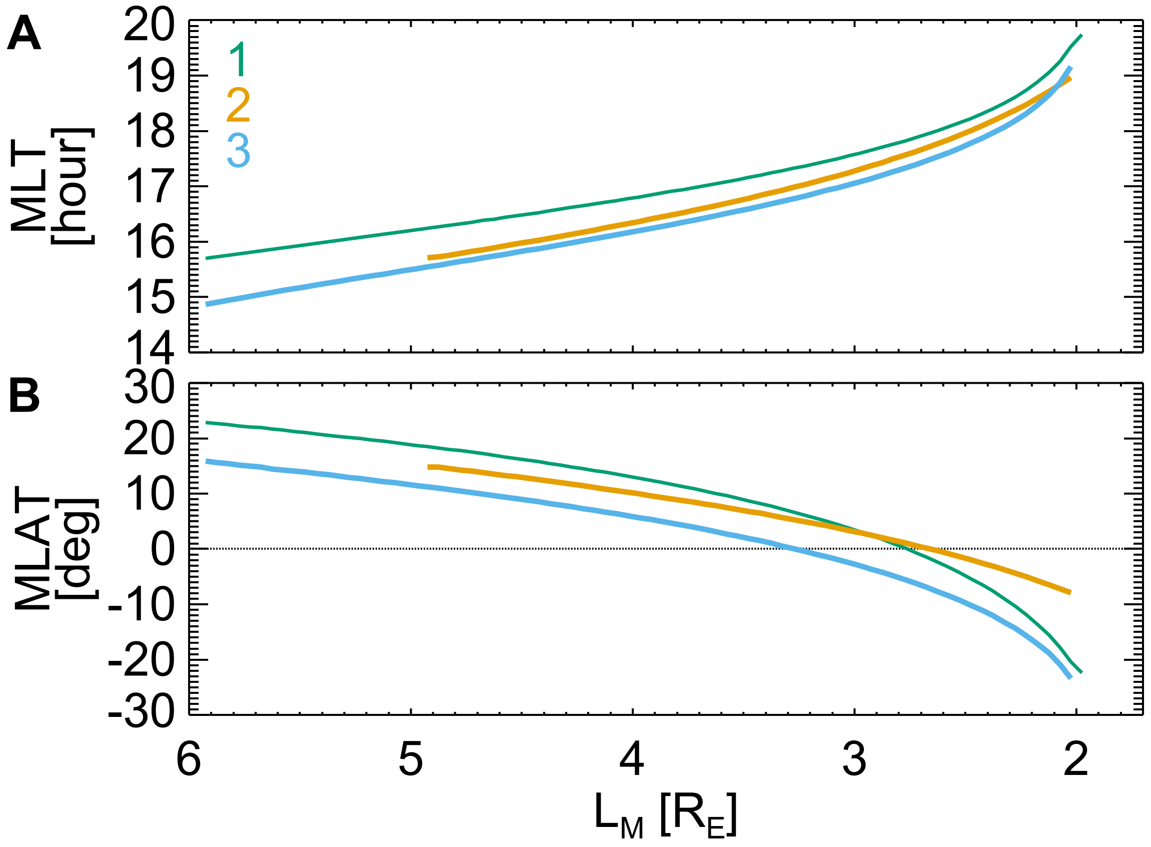

Fig. S1. L-shell (L_M) profiles of magnetic local time (MLT) and magnetic latitude (MLAT) of the Arase satellite. (**A**) MLT. (**B**) MLAT.

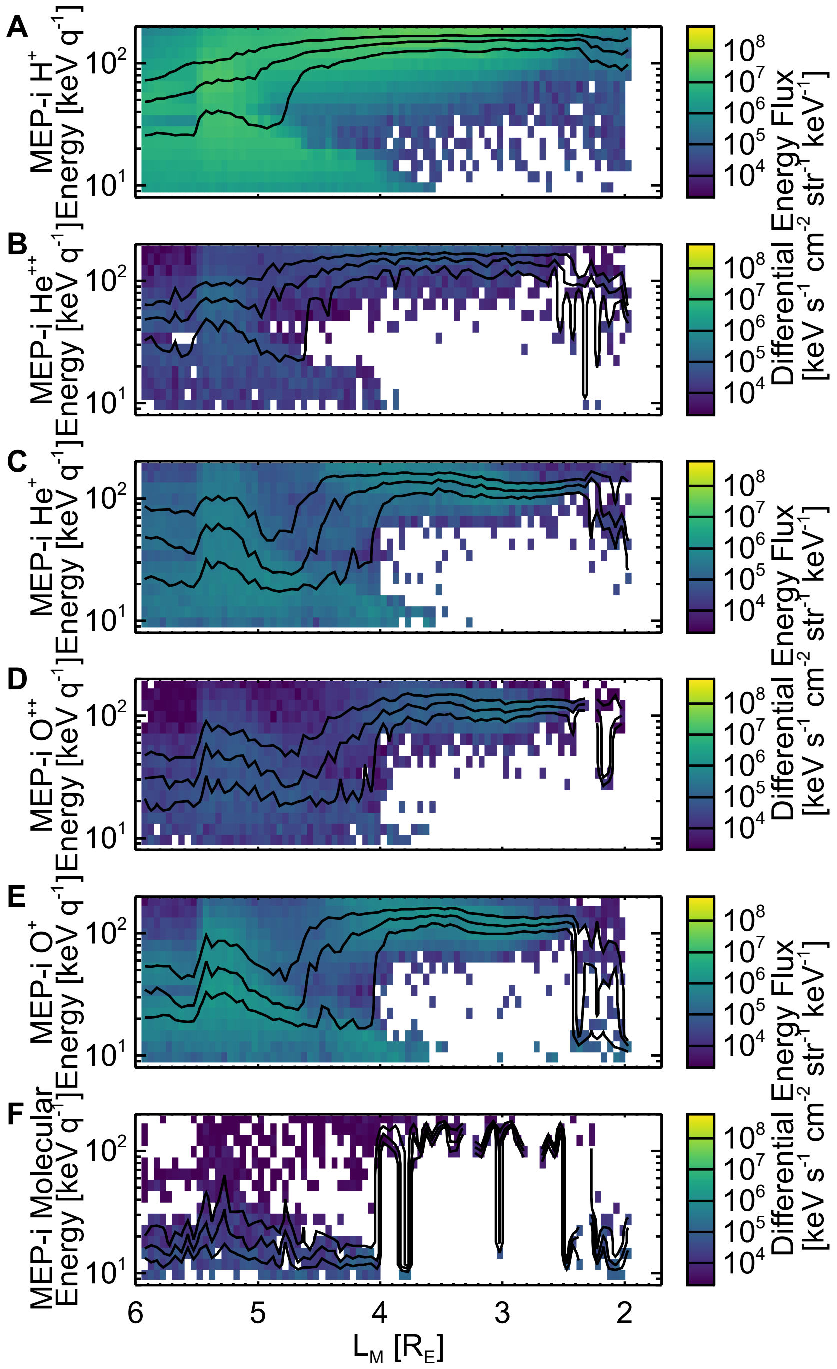

Fig. S2. Energy versus L-shell (L_M) spectra of differential energy fluxes of each ion species observed by the medium energy particle experiments-ion mass analyzer (MEP-i) (Orbit 1). (**A**) H^+. (**B**) He^{++}. (**C**) He^+. (**D**) O^{++}. (**E**) O^+. (**F**) Molecular ions (N_2^+, NO^+, and O_2^+). Black thin curves indicate the cumulative energy density ratio of 0.25, 0.5, and 0.75. Energy ranges between cumulative energy density ratios of 0.0 (lower energy limit) and 0.5, 0.25, and 0.75, or 0.5 and 1.0 (upper energy limit), which contain half of the total energy density of each ion species. The energy range between the ratios of 0.25 and 0.75 is a good indicator of the energy range that contributes dominantly if the spacing is narrow.

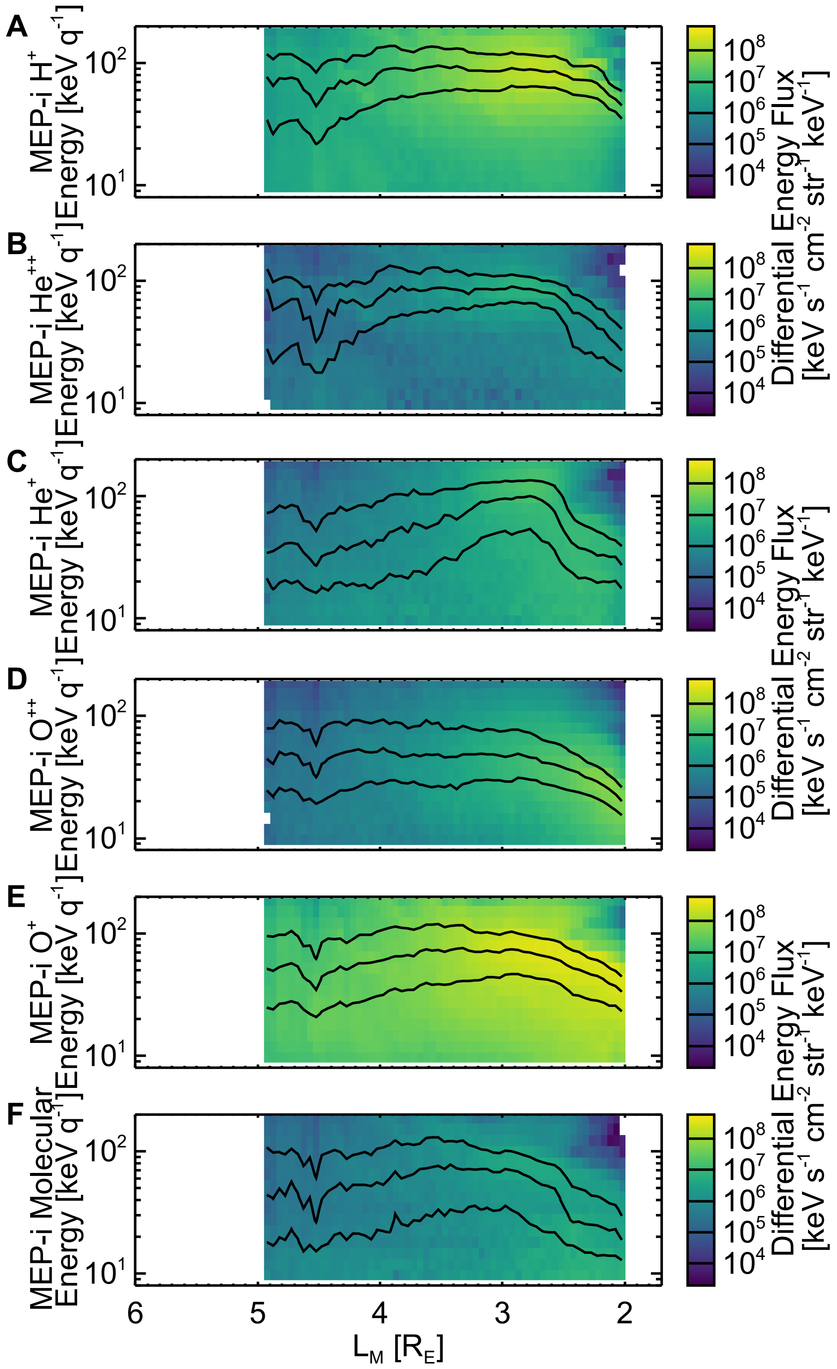

Fig. S3. Energy versus L-shell (L_M) spectra of differential energy fluxes of each ion species observed by the medium energy particle experiments-ion mass analyzer (MEP-i) (Orbit 2). The format is the same as that of Fig. S2.

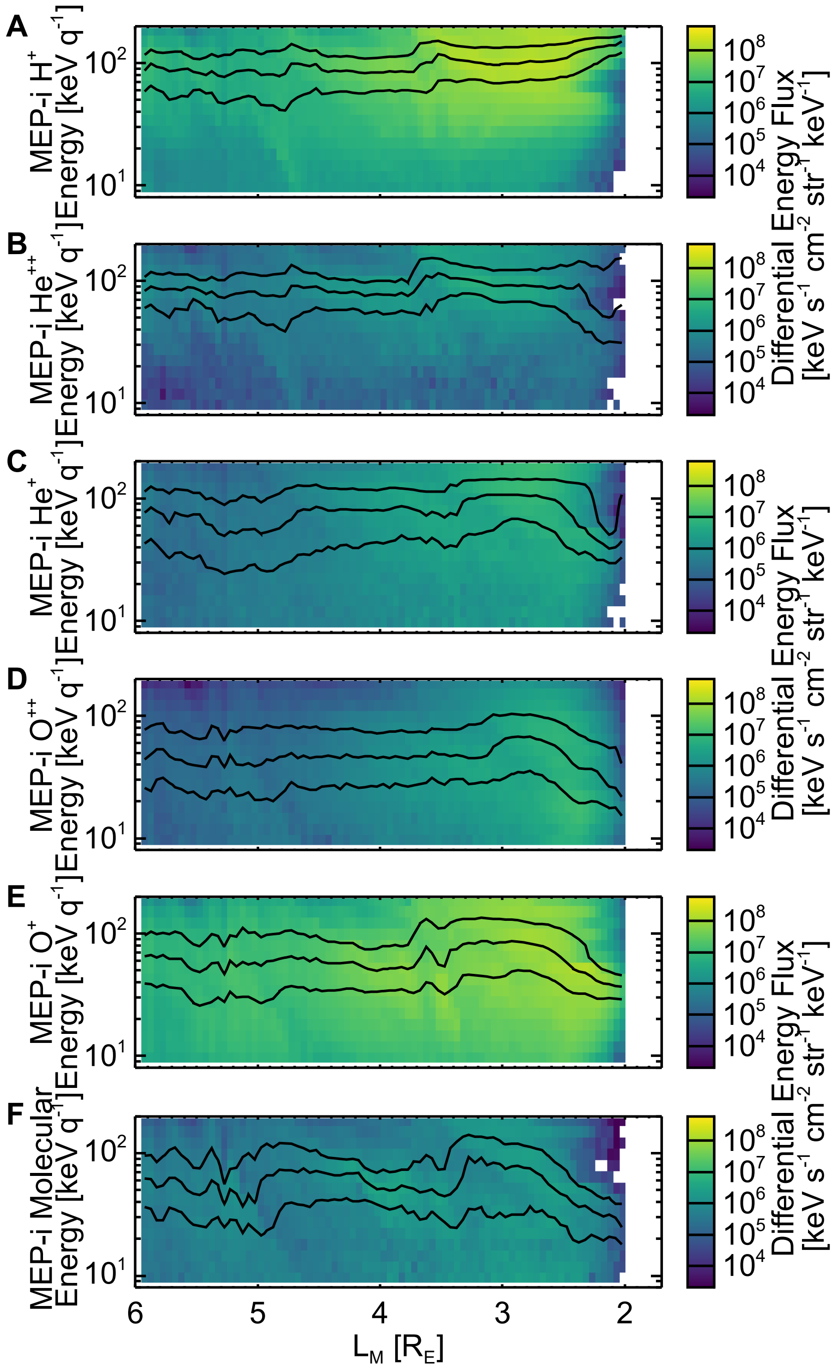

Fig. S4. Energy versus L-shell (L_M) spectra of differential energy fluxes of each ion species observed by the medium energy particle experiments-ion mass analyzer (MEP-i) (Orbit 3). The format is the same as that of Fig. S2.

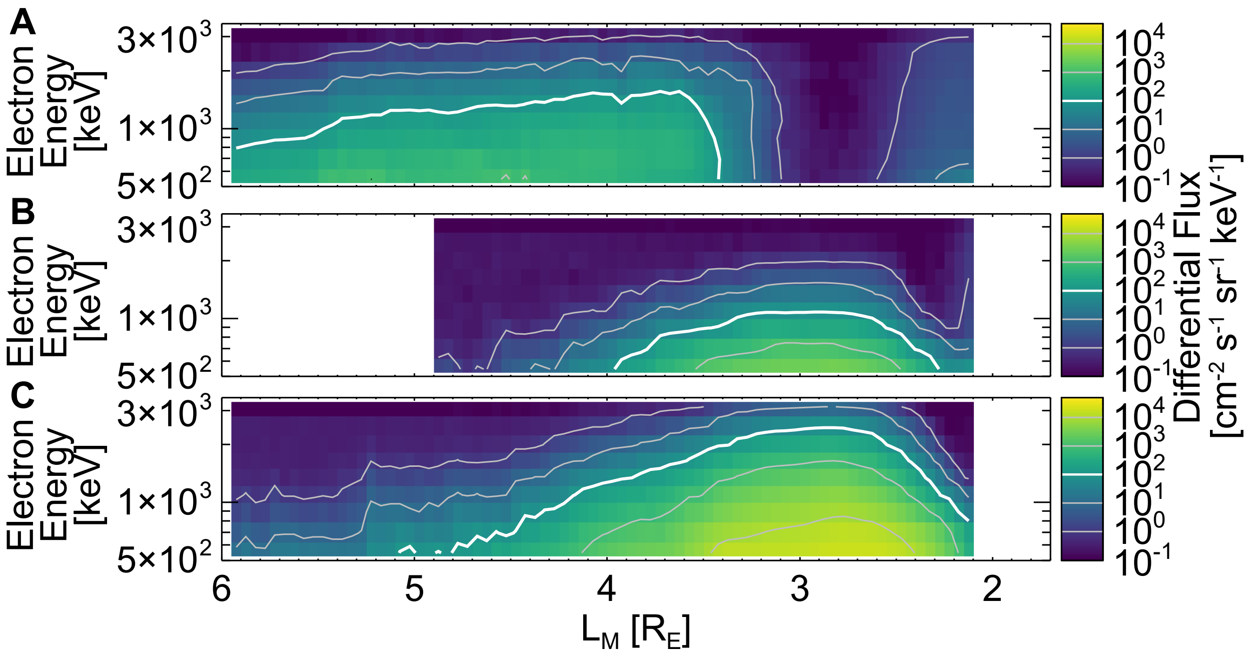

Fig. S5. Energy versus L-shell (L_M) spectra of differential fluxes of energetic electrons in the pitch angle range of 45°–135°, which was continuously covered by the extremely high energy electron experiment (XEP) during Orbits 1–3 in the magnetosphere. (**A**) Orbit 1. (**B**) Orbit 2. (**C**) Orbit 3.

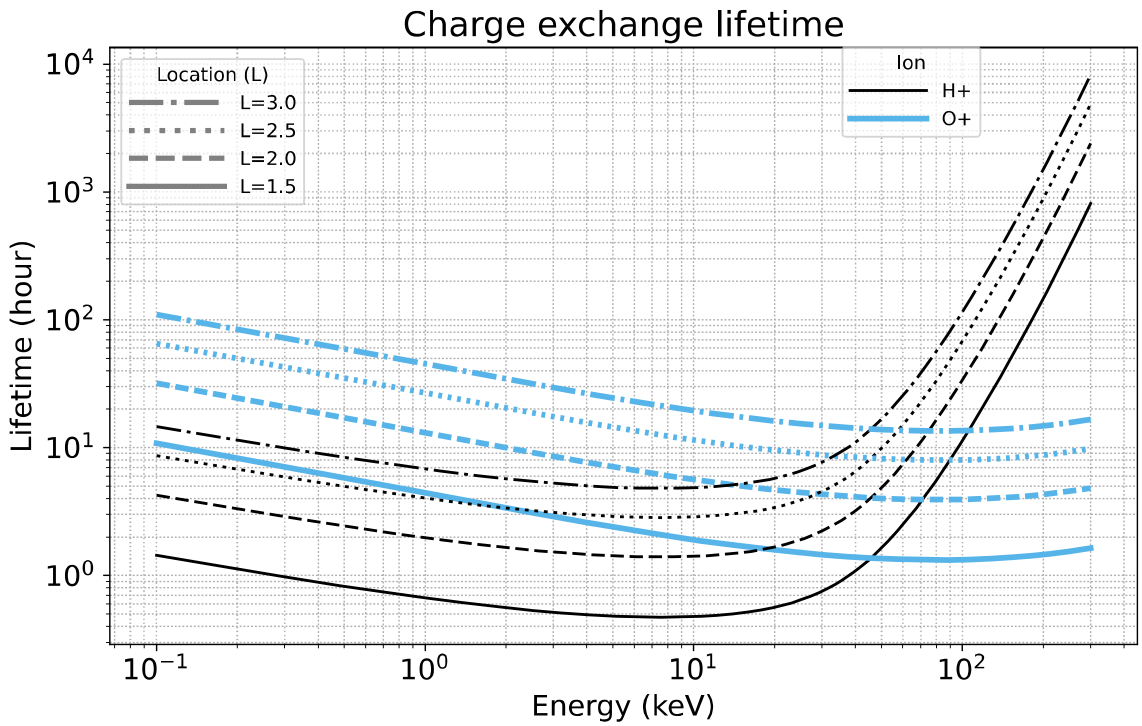

Fig. S6. Charge exchange lifetimes of equatorially-mirroring H^+ and O^+ near the Earth.
